# TXNRD1: A Key Regulator Involved in the Ferroptosis of CML Cells Induced by Cysteine Depletion In Vitro

**DOI:** 10.1155/2021/7674565

**Published:** 2021-12-07

**Authors:** Shuhan Liu, Wei Wu, Qiaoqian Chen, Zhiyuan Zheng, Xiandong Jiang, Yan Xue, Donghong Lin

**Affiliations:** ^1^Department of Laboratory Medicine, The School of Medical Technology and Engineering, Fujian Medical University, Fujian, China 350004; ^2^Medical Technology Experimental Teaching Center, The School of Medical Technology and Engineering, Fujian Medical University, Fujian, China 350004

## Abstract

Cysteine metabolism plays a critical role in cancer cell survival. Cysteine depletion was reported to inhibit tumor growth and induce pancreatic cancer cell ferroptosis. Nevertheless, the effect of cysteine depletion in chronic myeloid leukemia (CML) remains to be explored. In this work, we showed that cysteine depletion can induce K562/G01 but not K562 cell death in the form of ferroptosis. However, the glutathione (GSH)/glutathione peroxidase 4 (GPX4) pathways of the two CML cell lines were both blocked after cysteine depletion. This unexpected outcome guided us to perform RNA-Seq to screen the key genes that affect the sensitivity of CML cells to cysteine depletion. Excitingly, thioredoxin reductase 1 (*TXNRD1*), which related to cell redox metabolism, was significantly upregulated in K562/G01 cells after cysteine depletion. We further inferred that the upregulation is negatively feedback by the enzyme activity decrease of TXNRD1. Then, we triggered the ferroptosis by applying *TXNRD1* shRNA and TXNRD1 inhibitor auranofin in K562 cells after cysteine depletion. In summary, we have reason to believe that TXNRD1 is a key regulator involved in the ferroptosis of CML cells induced by cysteine depletion in vitro. These findings highlight that cysteine depletion serves as a potential therapeutic strategy for overcoming chemotherapy resistance CML.

## 1. Introduction

Chronic myeloid leukemia (CML) is a hematopoietic stem cell disease [1]. Although tyrosine kinase inhibitor imatinib can improve the prognostic survival rate of most CML patients, there are still many problems that hinder the therapeutic effect of CML, such as drug side effect and drug resistance [2–4]. Therefore, it is urgent to explore new strategies to overcome CML treatment bottleneck. Targeting cysteine metabolism is considered as a new potential therapy for cancers [5–7]. Research indicates that synthetic cysteine enzyme can decompose cysteine in the blood to suppress the tumor growth in breast and prostate cancer xenografts without obvious toxicity on normal cells [8]. It is speculated that targeting cysteine metabolic has broad application prospects.

As one of the most important amino acids, cysteine plays an irreplaceable role in maintaining the oxidation-reduction balance [9]. Cysteine provides active substance for the key molecule glutathione (GSH) in the redox reaction [10]. Most normal cells require very low levels of cysteine, but the demand of cysteine in cancer cells is very high [11]. It is fairly vital to the survival of cancer cells [12, 13]. Cancer cells accelerate their self-proliferation by increasing the metabolic rate, which leads to the accumulation of a great quantity of oxidation products, such as reactive oxygen species (ROS) [14]. Cancer cells must rely on the antioxidant defense system to avoid spontaneous death caused by oxidative burden. Therefore, disrupting this system by cysteine depletion to induce spontaneous oxidative death of cancer cells is a new breakthrough in the treatment of cancers.

Cysteine depletion can induce leukemia stem cell death by inhibiting the electron transport complex II, but has no effect on normal hematopoietic stem cells [15]. However, whether cysteine depletion can cause CML cell death and the form of the death is an open question. To explore this issue, in this present study, we create a cysteine-free culture environment for CML cells in vitro to observe cell viability; we found that cysteine depletion can induce CML cell death and confirmed that the form of death was ferroptosis. As a new form of cell death, ferroptosis is an oxidizing cell death that is different from apoptosis, necrosis, and autophagy [16]. Furthermore, we demonstrated that thioredoxin reductase 1 (*TXNRD1*) may be a key gene involved in the regulation of ferroptosis in CML cells. These findings suggest a potential cysteine depletion-based therapeutic strategy for CML to improve patient prognosis.

## 2. Materials and Methods

### 2.1. Cell Culture

The human CML line K562 (#CL-0130) and the imatinib-resistant K562 cell lines K562/G01 (#CL-0589) were purchased from the Procell Life Technology Company (Wuhan, China). The IC_50_ of K562 cells for imatinib is 0.147 ± 0.032 *μ*M, and the IC_50_ of K562/G01 cells for imatinib is 8.135 ± 1.767 *μ*M. The IC_50_ value of imatinib for K562/G01 cells was around 50-fold higher than that of K562. Cells were cultured in RPMI-1640 medium with 10% fetal bovine serum (FBS) (GEMINI, #A99G00K) at 37°C in an incubator with a humidified atmosphere of 5% CO_2_. The drug-resistant cell line K562/G01 cells were cultured in drug-free (imatinib) PRMI-1640 medium containing 10% FBS before the experiment. For cystine starvation experiments, cells were cultured in RPMI-1640 lacking glutamine, methionine, and cystine (Sigma, #R7513), supplemented with 200 mM methionine (Sigma, #M9625), 4 mM glutamine (Life Technologies, #25030081), and 10% FBS for the 24 h.

### 2.2. Reagents

Ferrostatin-1(#GC10380), necrostatin-1s (#GC11008), Z-VAD-FM (#GC12861), chloroquine (#GC10295), and auranofin (#GC15905) were purchased from GlpBio.

### 2.3. Cell Viability Assay

Cell viability was measured using MTS cell proliferation colorimetric assay kit (Promega, #G3580). K562 and K562/G01 cells were seeded into 96-well plates and exposed to conditions or treated with reagents as indicated at 24 h. Subsequently, 20 *μ*L of the MTS solution was added to each well of the plate and incubated for 4 h at 37°C, 5% CO_2_. Then, the absorbance (OD value) at wavelengths of 490 nm was measured with a microplate reader (Thermo, #1410101).

### 2.4. Cell Ultrastructure Assay

The treated cells were fixed and put into alcohol and acetone for dehydration, and the specimens were embedded in epoxy resin. The Leica UC-7 microtome cuts ultrathin sections of 90-100 nm and dyes the sections with uranyl acetate. Finally, the film was observed and taken under the TECNAI transmission electron microscopy (TEM) of FEI Company (USA).

### 2.5. Lipid Peroxidation Assay

The MDA level in cells was measured using a Micro Malondialdehyde Assay Kit (#BC0025). We followed the methods of Gao et al. (2021) [17]. Cells were homogenized with lysis buffer, and the MDA in samples reacts with thiobarbituric acid (TBA) to generate an MDA-TBA adduct which can be quantified colorimetrically (OD = 532 nm).

BODIPY™ 581/591 C11 (Invitrogen, #D3861) can be used to detect ROS in cells. Treated cells were incubated with 1 *μ*M BODIPY™ 581/591 C11 for 30 minutes. After incubation, the cells were washed with phosphate-buffered saline (PBS) and analyzed by the Accuri C6 Flow Cytometer (BD, USA).

### 2.6. Redox Metabolism Assay

The redox metabolites were measured using Micro Reduced Glutathione Assay Kit (Solarbio, #BC1175), Glutathione Peroxidase 4 Assay Kit (Bioss, #AK091), and Micro Oxidized Thioredoxin Reductase Assay Kit (Solarbio, #BC1155). And the experiments were carried out according to the manufacturer's instructions. Cells were homogenized with lysis buffer, and the redox substances in samples react with reagent to generate adduct which can be quantified colorimetrically.

### 2.7. RNA-Seq

K562/G01 cells were cultured in normal (control) or cysteine depletion (treat) condition for 24 h. The total RNA of the above two groups was isolated using TRIzol (CWBIO, #CW0580S) following the manufacturer's instructions. Qualified RNAs were finally quantified by Qubit3.0 with Qubit™ RNA Broad Range Assay kit (Life Technologies, USA) and used for stranded RNA sequencing library preparation using KC™ Stranded mRNA Library Prep Kit for Illumina® (Wuhan Seqhealth Inc., China). PCR products corresponding to 200-500 bp were enriched, quantified, and finally sequenced on Hiseq X10 sequencer (Illumina, USA).

### 2.8. Quantitative Real-Time Polymerase Chain Reaction Assay (qRT-PCR)

Total RNA was extracted using TRIzol. cDNA was synthesized from 1 *μ*g total RNA using the Hifair TMII First-Strand cDNA Synthesis Super Mix (Yeasen, #11123ES60). The experiment was performed for reverse transcription according to the methods of Gao et al. (2021) [17]. Subsequently, SYBR Green-based real-time PCR was performed in triplicate using SYBR Green master mix (Yeasen, #11202ES08) on a 7500 real-time fluorescent quantitative PCR machine (ABI, USA). For analysis, the threshold cycle (Ct) values for each gene were normalized to expression levels of *β*-actin. All primers were designed and synthesized by Shangya Biotechnology. The primer sequences used in PCR are as follows: *TXNRD1*: 5′-TCACAGATGAAGAACAGACCAATG-3′ (forward), 5′-GCCACAAGCACCATATTCCA-3′ (reverse) and *β*-actin: 5′-TGACGTGGACATCCGCAAAG-3′ (forward), 5′-CTGGAAGGTGGACAGCGAGG-3′ (reverse).

### 2.9. Western Blot Analysis

To evaluate the changes in protein expression by Western blot, we followed the methods of Chang et al. (2021) [18]. Briefly, cells were lysed with lysis buffer and centrifuged at 4°C. The protein concentration was determined by a bicinchoninic acid (BCA) protein assay kit (Sigma, #71285-M). Protein lysates (40 *μ*g) were separated by sodium dodecyl sulfate-polyacrylamide gel electrophoresis (SDS-PAGE) and electrotransferred to polyvinylidene difluoride (PVDF) membranes. The membranes were blocked with 5% nonfat milk in TBST buffer (TBS buffer containing 0.1% Tween 20) for one hour and incubated with primary antibodies such as TXNRD1 (ImmunoWay, #YN2811) and *β*-actin (Bioss, #bs-0061R) as well as HRP-conjugated secondary antibodies. HRP luminescence was detected with an enhanced chemiluminescence (ECL) detection kit (Meilunbio, #MA0186).

### 2.10. RNAi and Gene Transfection

Cancer cells were seeded in 6-well plates to achieve a confluence of 60-70% overnight. To generate knockdown cells, cells were infected with lentivirus carrying shRNA followed by puromycin (1.5 *μ*g/mL) selection for 5-7days. These established stable cell lines were maintained in RPMI-1640 containing 10% FBS and puromycin (1 *μ*g/mL) for further experiments. The specific *TXNRD1* shRNA sequences are follows: 5′-ATGTTCCAACCACTGTATTTA-3′. NC shRNA sequences are follows: 5′-UUCUCCGAACGUGUCACGUTT-3′.

### 2.11. Statistical Analysis

All data are presented as the mean ± standard error of the mean (SEM). The differences between groups were analyzed using Student's *t*-tests or one-way analysis of variance (ANOVA). *P* < 0.05 was considered to reflect a statistically significant difference. All the experiments were repeated at least three times.

## 3. Results

### 3.1. Cysteine Depletion Inhibits the Cell Viability of K562/G01 but Not K562

**Figure 1:**
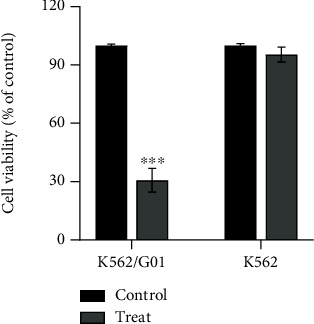
Cysteine depletion inhibits the cell viability of K562/G01 but not K562. K562/G01 and K562 cells were cultured in normal or cysteine depletion condition for 24 h. Cell viability was measured using the MTS kit. ^∗∗∗^*P* < 0.001 vs. control group.

Adequate cysteine helps cancer cells maintain redox balance and avoid oxidative death [14, 19]. We firstly access the effect of cysteine depletion on CML cell viability. To mimic the cysteine depletion condition in vitro, K562 and K562/G01 cells were cultured with cysteine-free RPMI-1640 with 10% FBS for 24 h. MTS showed that the cell viability of K562/G01 was decreased after cysteine depletion (treat) compared with normal culture condition (control), while there is no change in K562 cells (Figure 1, *P* < 0.001).

### 3.2. Cysteine Depletion Induces Ferroptosis in K562/G01 but Not K562 Cells

**Figure 2:**
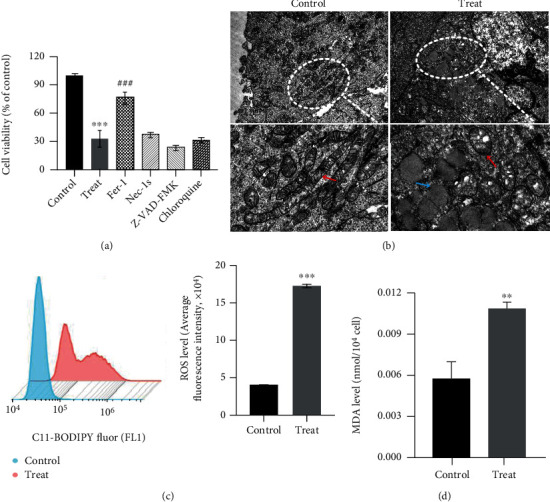
Cysteine depletion induces ferroptosis in K562/G01 but not K562 cells. (a) K562/G01 cells were treated with Fer-1 (2 *μ*M), Nec-1s (10 *μ*M), Z-VAD-FMK (10 *μ*M), or chloroquine (25 *μ*M) cultured in normal or cysteine depletion condition for 24 h. Cell viability was measured using the MTS kit. (b) TEM images showed the cell ultrastructure changes of K562/G01 cells cultured in normal or cysteine depletion condition for 24 h (scale bar = 1 *μ*m). (c) Flow cytometry detected the ROS level of K562/G01 cells cultured in normal or cysteine depletion condition for 24 h. (d) The levels of MDA were tested in K562/G01 cells cultured in normal or cysteine depletion condition for 24 h. Fer-1 represents for ferrostatin-1; Nec-1s represents for necrostatin-1s; control represents for normal condition (0.0652 g/L cysteine). Treat represents for cysteine depletion condition (0 g/L cysteine). Red arrow: mitochondria. Blue arrow: lipid droplets. Experiments were repeated three times, and the data are expressed as the mean ± SEM. ^∗∗^*P* < 0.01 vs. control group, ^∗∗∗^*P* < 0.001 vs. control group, and ^###^*P* < 0.001 vs. treat group.

In order to further clarify the form of CML cell death, four different cell death inhibitors including ferroptosis, necrosis, apoptosis, and autophagy were used to treat CML cells. The inhibition of the cell viability of K562/G01 after cysteine depletion could be reversed by the ferroptosis inhibitor (Fer-1) only (Figure 2(a), *P* < 0.001). Ferroptosis, a new regulated form of cell death, is genetically, biochemically, and morphologically distinct from above other cell death forms [20]. The morphological characteristics of ferroptosis mainly include mitochondria cristae decreased or vanished, outer mitochondrial membrane ruptured, and mitochondrial membrane condensed [21]. So, we observed the morphological changes of CML cells by TEM; the ultrastructural changes of K562/G01 cells after cysteine depletion are consistent with the abovementioned characteristic changes of ferroptosis (Figure 2(b)). These cell abnormalities resulted from the occurrence of oxidative stress due to subsequent accumulation of lipid peroxidation, particularly lipid-based ROS and malondialdehyde (MDA) [22, 23]. Then, we detected ROS and MDA levels and found that the two lipid peroxide indexes in K562/G01 cells increased after cysteine depletion (Figures 2(c) and 2(d), *P* < 0.01). The above phenomena were not observed in K562 cells (Supplementary Figure 1). Therefore, these results indicated that cysteine depletion can induce ferroptosis in K562/G01 cells but not K562.

### 3.3. Cysteine Depletion Blocks the GSH/GPX4 Pathway in Both K562/G01 and K562 Cells

**Figure 3:**
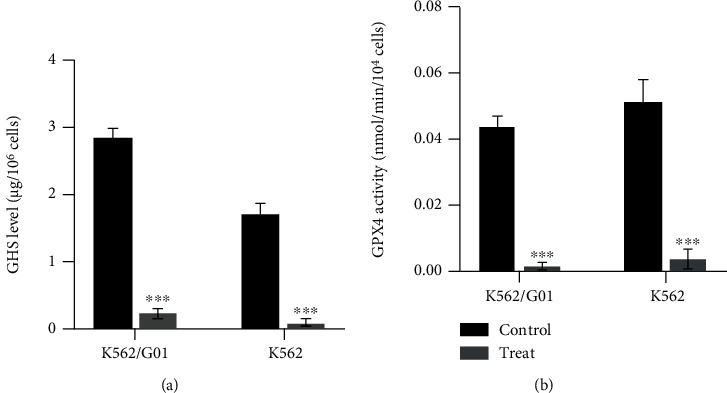
Cysteine depletion blocks the GSH/GPX4 pathway in both K562/G01 and K562 cells. (a) The levels of GSH were tested in K562/G01 and K562 cells cultured in normal or cysteine depletion condition for 24 h. (b) The activity of GPX4 was tested in K562/G01 and K562 cells cultured in normal or cysteine depletion condition for 24 h. GSH represents for reduced glutathione; GPX4 represents for glutathione peroxidase 4; control represents for normal condition (0.0652 g/L cysteine). Treat represents for cysteine depletion condition (0 g/L cysteine). Experiments were repeated three times, and the data are expressed as the mean ± SEM. ^∗∗∗^*P* < 0.001 vs. control group.

Studies demonstrated that GSH/GPX4 (glutathione peroxidase 4) is a classical regulation pathway of ferroptosis [24]. GSH/GPX4 axis can convert lipid hydroperoxides to lipid alcohols, and this process prevents the formation and accumulation of toxic lipid ROS [25, 26]. To clarify the pathway changes in CML cells after cysteine depletion, we tested the GSH level and GPX4 activity in CML cells. Both of them in K562/G01 cells significantly decreased after cysteine depletion (Figures 3(a) and 3(b), *P* < 0.001). These results revealed that GSH/GPX4 axis is involved in regulating the ferroptosis of K562/G01 cells induced by cysteine depletion. Surprisingly, although K562 cells did not occur ferroptosis, their GSH level and GPX4 activity showed the same changes as K562/G01.

### 3.4. Cysteine Depletion Can Obviously Feedback Increase TXNRD1 Expression in K562/G01 Cells

**Figure 4:**
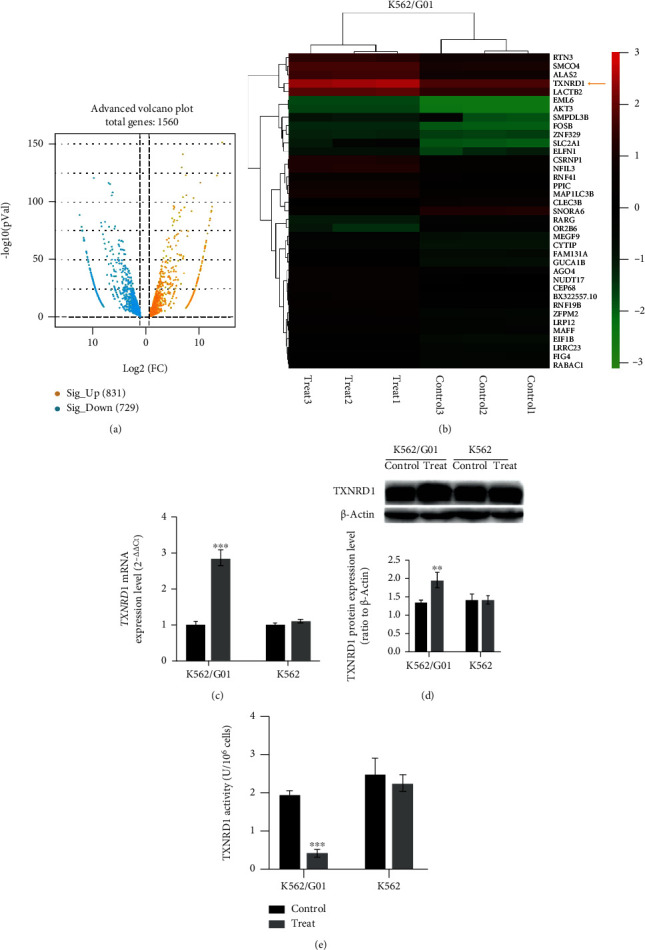
Cysteine depletion can obviously feedback increase *TXNRD1* expression in K562/G01 cells. (a) Volcano map of differential genes of K562/G01 between the control group and the cysteine depletion group. (b) Heat map of differential gene cluster analysis. Each column represents an experimental sample, and each row represents a gene. Expression differences are shown in different colors. Red means high expression and green means low expression. (c) *TXNRD1* mRNA expression level of K562/G01 and K562 cells cultured in normal or cysteine depletion condition for 24 h. (d) TXNRD1 protein expression level of K562/G01 and K562 cells cultured in normal or cysteine depletion condition for 24 h. (e) The activity of TXNRD1 was tested of K562/G01 and K562 cells cultured in normal or cysteine depletion condition for 24 h. Control represents for normal condition (0.0652 g/L cysteine). Treat represents for cysteine depletion condition (0 g/L cysteine). Experiments were repeated three times, and the data are expressed as the mean ± SEM. ^∗∗^*P* < 0.01 vs. control group and ^∗∗∗^*P* < 0.001 vs. control group.

To further clarify the different ferroptosis sensitivity of CML cells after cysteine depletion and the molecular mechanism, we used RNA-Seq to detect differentially expressed genes of K562/G01 cells after cysteine depletion. The results showed that 831 genes were upregulated and 729 genes were downregulated in K562/G01 cells after cysteine depletion (log_2_(FC) > 1 or < -1, Figure 4(a)). Gene cluster analysis showed that *TXNRD1* is one of the upregulated genes (Figure 4(b)). Then, qRT-PCR and Western blot were performed to confirm the expression changes of TXNRD1 mRNA and protein in CML cells after cysteine depletion. The results showed that the *TXNRD1* gene expression in K562/G01 cells was remarkably elevated after cysteine depletion (*P* < 0.01), while there were no changes in K562 cells (*P* > 0.05) (Figures 4(c) and 4(d)). *TXNRD1* gene can encode thioredoxin reductase 1 (TXNRD1), which is a critical antioxidant selenoprotein enzyme that regulates cellular redox homeostasis [27]. Therefore, we sought to further evaluate the TXNRD1 activity of CML cells after cysteine depletion. In K562/G01 cells, TXNRD1 activity did not increase with *TXNRD1* gene upregulation after cysteine depletion. On the contrary, TXNRD1 activity decreased significantly; this phenomenon may be caused by negative feedback. While TXNRD1 activity has no change in K562 cells, the result was consistent with *TXNRD1* gene expression (Figure 4(e)). These data suggested that TXNRD1 may be closely related to the ferroptosis sensitivity of CML cells.

### 3.5. Cysteine Depletion Induces K562 Cell Ferroptosis after Downregulating TXNRD1

**Figure 5:**
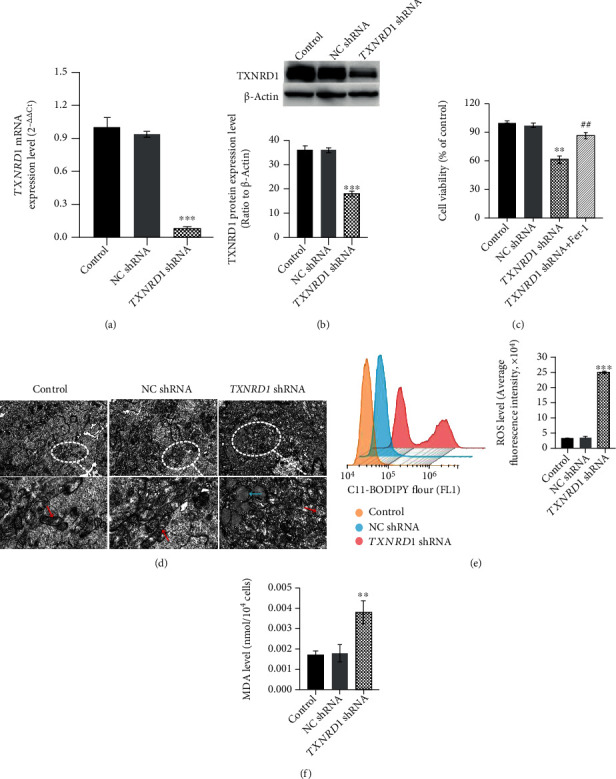
Cysteine depletion induces K562 cell ferroptosis after downregulating *TXNRD1*. (a, b) qRT-PCR and Western blot analyzed the shRNA-mediated knockdown of *TXNRD1* mRNA and protein expression levels in K562 cells. *β*-Actin mRNA and protein expressions were detected as a loading control for qRT-PCR and Western blot. (c) Cell viability was measured in NC shRNA and *TXNRD1* shRNA K562 cells cultured in cysteine depletion condition for 24 h. (d) TEM images showed the cell ultrastructure changes of NC shRNA and *TXNRD1* shRNA K562 cells cultured in cysteine depletion condition for 24 h (scale bar = 2 *μ*m). (e, f) ROS and MDA levels of NC shRNA or *TXNRD1* shRNA K562 cells cultured in cysteine depletion condition were tested. ^∗^*P* < 0.05 vs. control group, ^∗∗^*P* < 0.01 vs. control group, ^∗∗∗^*P* < 0.001 vs. control group, and ^##^*P* < 0.01 vs. *TXNRD1* shRNA group.

To further explore the role of *TXNRD1* in cysteine depletion-induced K562 cell ferroptosis, we established stable knockdown cell clone (*TXNRD1* shRNA) and verified the silencing efficiency by qRT-PCR and Western blot (Figures 5(a) and 5(b), *P* < 0.001). The effect of *TXNRD1* downregulation on the cell viability, morphology and lipid oxidation of K562 cells was detected after cysteine depletion. Compared to the NC shRNA group, the cell viability of the *TXNRD1* shRNA group was significantly decreased after cysteine depletion, which was reversed in the presence of Fer-1 (Figure 5(c), *P* < 0.01). These exciting results had emerged in K562 cells after downregulating *TXNRD1*; the TEM showed that the mitochondrial cristae decreased, mitochondrial outer membrane ruptured, and lipid droplets accumulated in the cytoplasm after cysteine depletion (Figure 5(d)). In addition, the ROS and MDA levels in the *TXNRD1* shRNA group increased after cysteine depletion (Figures 5(e) and 5(f), *P* < 0.01). The above observations suggest that cysteine depletion induces K562 cell ferroptosis after downregulating *TXNRD1*.

### 3.6. Cysteine Depletion Induces K562 Cell Ferroptosis after Inhibiting the Activity of TXNRD1

**Figure 6:**
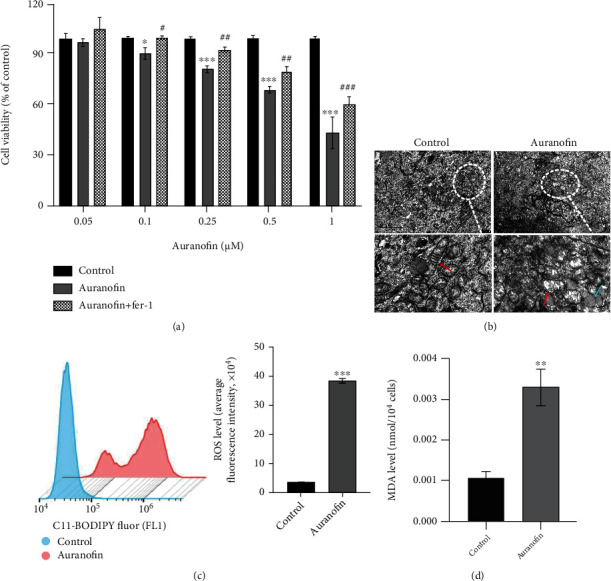
Cysteine depletion induces K562 cell ferroptosis after inhibiting the activity of TXNRD1. (a) Cell viability of K562 cells treated with auranofin or auranofin and Fer-1 and cultured in cysteine depletion condition was measured using the MTS kit. (b) TEM images showed the cell ultrastructure changes of K562 cells treated with auranofin under cysteine depletion condition (scale bar = 2 *μ*m). (c, d) ROS and MDA levels of K562 cells treated with auranofin under cysteine depletion condition were measured. Auranofin: 1 *μ*M. ^∗^*P* < 0.05 vs. control group, ^∗∗^*P* < 0.01 vs. control group, ^∗∗∗^*P* < 0.001 vs. control group, ^#^*P* < 0.05 vs. auranofin group, ^##^*P* < 0.01 vs. auranofin group, and ^###^*P* < 0.001 vs. auranofin group.

Whether the activity of TXNRD1 affects the ferroptosis sensitivity of K562 cells induced by cysteine depletion is unknown. For further confirmation, TXNRD1 inhibitor (auranofin) was used to treat K562 cells cultured in cystine-free RPMI-1640. Then, we tested ferroptosis-related indicators. As expected, the K562 cells treated with auranofin showed obvious ferroptosis characteristics after cysteine depletion in accordance with the above results of *TXNRD1* downregulation (Figure 6). Taken together, these results suggest that *TXNRD1* may be a key gene involved in the regulation of ferroptosis in CML cells.

## 4. Discussion

Ferroptosis is a new form of cell death proposed by Dixon in 2012 that is different from apoptosis, necrosis, and autophagy [16]. Ferroptosis is closely related to a variety of cancers, such as hepatocellular carcinoma, gastric cancer, and breast cancer [28–30]. Several reports have shown the potential of triggering ferroptosis for cancer therapy, particularly for eradicating aggressive malignancies that are resistant to traditional therapies [31]. As mentioned in the literature, the potential for ferroptosis as a therapy for pancreatic tumor has been tested in vivo and in vitro trials; cysteine depletion can induce pancreatic cancer cell ferroptosis to inhibit the tumor growth [32]. In the current study, we cultured two CML cell lines with cystine-free medium and tested the cell viability by MTS. The results showed cysteine depletion can obviously suppressed the cell viability of K562/G01 cells. Then, we further treated K562/G01 using 4 cell death inhibitors to clarify the form of cell death. What is surprising is that only ferroptosis inhibitor can significantly rescue the suppression of the K562/G01 cell viability after cysteine depletion. TEM results showed that K562/G01 cells occurred ultrastructural changes after cysteine depletion, such as mitochondrial cristae disappeared, the membrane was incomplete, and there were a large number of lipid droplets in the cytoplasm. The abovementioned ultrastructural changes are characteristic changes of ferroptosis [33]. In addition, changes in biochemical indicators related to ferroptosis mainly focus on lipid peroxidation, including ROS and MDA [16]. Hence, we detected changes of ROS and MDA levels in K562/G01 cells; both of them were significantly increased after cysteine depletion. High levels of ROS will cause cancer cell oxidative damage and even spontaneous death, and the accumulation of oxidation products is one of the necessary conditions for the occurrence of ferroptosis [14]. The above results gave us an affirmative answer whether cysteine depletion will cause CML cell death and the form of cell death: cysteine depletion can induce K562/G01 cell ferroptosis.

Previous research points out that the GSH/GPX4 axis involved in lipid peroxidation metabolism is a classic way to regulate ferroptosis [24]. High-level antioxidant defense key molecule GSH can prevent cancer cells from dying due to oxidative stress, and the active substance of GSH is provided by cysteine [10]. GPX4 is an important peroxidase decomposing enzyme widely distributed in the body, which is one of the main enzymes that catalyze the oxidation of GSH in the glutathione redox cycle [34]. In the condition of cysteine depletion, the synthesis of GSH in cancer cells is blocked, the activity of GPX4 is reduced, and the redox imbalance in the cell leads to a large accumulation of lipid peroxides and eventually ferroptosis [19, 35]. Therefore, we detected the changes in GSH level and GPX4 activity of CML cells after cysteine depletion. The results showed that cysteine depletion can reduce the GSH level and GPX4 activity of K562/G01 cells, which indicates that GSH/GPX4 was involved in ferroptosis induced by cysteine depletion in K562/G01 cells. However, it is worth mentioning that ferroptosis was absent in another CML cell line K562 after cysteine depletion. Contrary to expectation, GSH level and GPX4 activity were significantly decreased even if there was no ferroptosis occurred in K562 cells after cysteine depletion. The difference between K562/G01 and K562 aroused our research interest. Were there other key factors that regulated the ferroptosis of CML cells?

In order to resolve this issue, we subsequently screened the differentially expressed genes of CML cell line K562/G01 by RNA-Seq, which can occur ferroptosis after cysteine depletion. We found that *SLC2A1*, *ZFPM2*, *NFIL3*, *TXNRD1*, *AKT3*, etc. were significantly upregulated after cysteine depletion. Interestingly, Yang et al. [36] reported that *TXNRD1* gene can regulate ferroptosis of liver cancer cells. *TXNRD1* has attracted our attention. Then, we used qRT-PCR and Western blot to verify the TXNRD1 mRNA and protein expression levels, and the results showed that the *TXNRD1* gene expression of K562/G01 cells in the cysteine depletion group was increased. *TXNRD1* gene encodes the thioredoxin reductase 1 (TXNRD1), and the active substance of TXNRD1 is provided by cysteine [37, 38]. Several studies have shown that the thioredoxin system is a key antioxidant system in defense against oxidative stress through its disulfide reductase activity regulating protein dithiol/disulfide balance. TXNRD1, as one of the important components of the thioredoxin system, plays a key role in protecting cells against oxidative damage [39–41]. Therefore, we speculate that cysteine depletion can directly cause the decrease of TXNRD1 activity in K562/G01 cells, leading to the imbalance of lipid peroxidation metabolism, which resulted in ferroptosis of K562/G01 cells. In present study, we tested the activity of TXNRD1 using micromethod. Unsurprisingly, the TXNRD1 activity of K562/G01 cells was significantly reduced after cysteine depletion. However, the decrease in TXNRD1 activity is opposite to the upregulation of *TXNRD1*, which can be considered as a resistance to ferroptosis. To maintain the level of TXNRD1 enzyme activity, *TXNRD1* gene expression was increased in K562/G01 cells. Despite such increase, it cannot reverse the decrease in enzyme activity caused by the lack of active substance to change the outcome of ferroptosis. These results suggest that the decrease of TXNRD1 activity may cause cells to negatively feedback upregulation of the expression of its coding gene *TXNRD1*.

It is worth noting that there were no significant changes in TXNRD1 mRNA and protein levels and TXNRD1 activity in CML cell line K562. This may be the reason why the ferroptosis has not been observed in K562 cells after cysteine depletion. In order to investigate whether *TXNRD1* is a key gene involved in regulating ferroptosis of CML cells, we used *TXNRD1* shRNA to downregulate the expression of *TXNRD1* gene. Excitingly, downregulating *TXNRD1* can really cause ferroptosis in K562 cells after cysteine depletion. Coincidentally, this result was also confirmed in subsequent experiments to inhibit TXNRD1 activity with auranofin. It should be emphasized that although cysteine depletion can reduce the activity of the GSH/GPX4 pathway in K562 cells, it seems that the changes of GSH/GPX4 still not enough to induce ferroptosis after cysteine depletion. When we downregulate the expression of *TXNRD1* or inhibit TXNRD1 activity, a large amount of ROS accumulates results in ferroptosis characteristic ultrastructural changes in K562 cells after cysteine depletion. This suggests that in CML cells, the occurrence of ferroptosis may require the coordinated regulation of the two antioxidant defense systems, TXNRD1 and GSH/GPX4. Therefore, it is reasonable to believe that TXNRD1 is a highly likely key regulator of the ferroptosis in CML cells.

## 5. Conclusions

**Figure 7:**
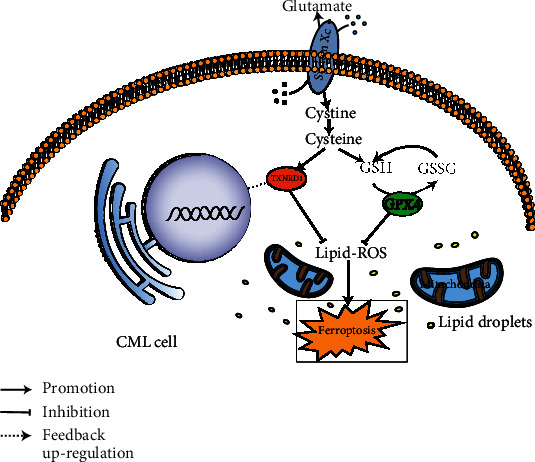
Schematic representation of the mechanisms of TXNRD1 regulates ferroptosis induced by cysteine depletion in CML cells. When the intracellular cysteine is sufficient, TXNRD1 and GSH/GPX4 work together to maintain the intracellular redox balance, avoiding cell oxidative damage and even ferroptosis. When intracellular cysteine is deficient, cysteine depletion will cause a certain degree of TXNRD1 enzyme activity and GSH/GPX4 levels decrease, leading to a sharp increase of ROS levels and the occurrence of ferroptosis, which negatively feedback upregulates the expression of *TXNRD1* gene.

In summary, this study reveals that cysteine depletion can induce ferroptosis in CML cells and TXNRD1 may be a key regulator gene (Figure 7). This illustrates that cysteine metabolism-induced ferroptosis may be a new idea for the treatment of CML except chemotherapy, which deserves further study. In the future, we will verify the ferroptosis induction effect of cysteine depletion on CML through in vivo experiments and explore the molecular regulation mechanism of TXNRD1 in ferroptosis. Our findings are expected to provide a new targeting cysteine metabolic therapy strategy to improve CML patient prognosis.

## Data Availability

The data used to support the findings of this study are available from the corresponding authors upon request.

## Supplementary Materials

Supplementary Materials Supplementary Figure 1: cysteine depletion cannot induce ferroptosis in K562 cells. (a) K562 cells were treated with Fer-1 (2 *μ*M), necrostatin-1s (10 *μ*M), Z-VAD-FMK (10 *μ*M), or chloroquine (25 *μ*M) cultured in normal or cysteine depletion condition for 24 h. Cell viability was measured using the MTS kit. (b) TEM observed the cell ultrastructure changes of K562 cells cultured in normal or cysteine depletion condition for 24 h (scale bar = 1 *μ*m). (c) Flow cytometry tested the ROS level of K562 cells cultured in normal or cysteine depletion condition for 24 h. (d) The levels of MDA were tested in K562 cells cultured in normal or cysteine depletion condition for 24 h.
