# Supplementary figures and images for: TXNRD1: A Key Regulator Involved in the Ferroptosis of CML Cells Induced by Cysteine Depletion In Vitro
Source: Oxid Med Cell Longev. 2021 Dec 7;2021:7674565. doi: 10.1155/2021/7674565 (PMC8670935; doi:10.1155/2021/7674565)

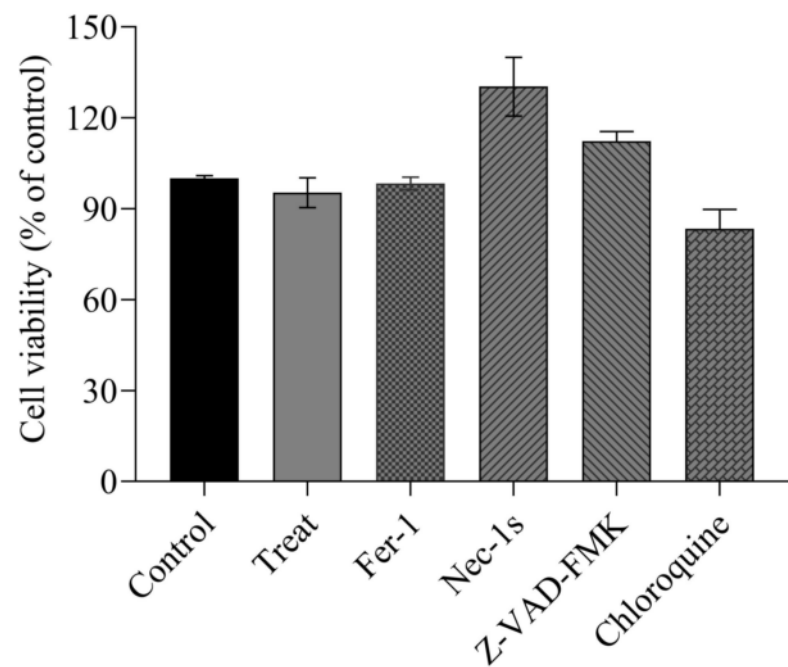

(a)

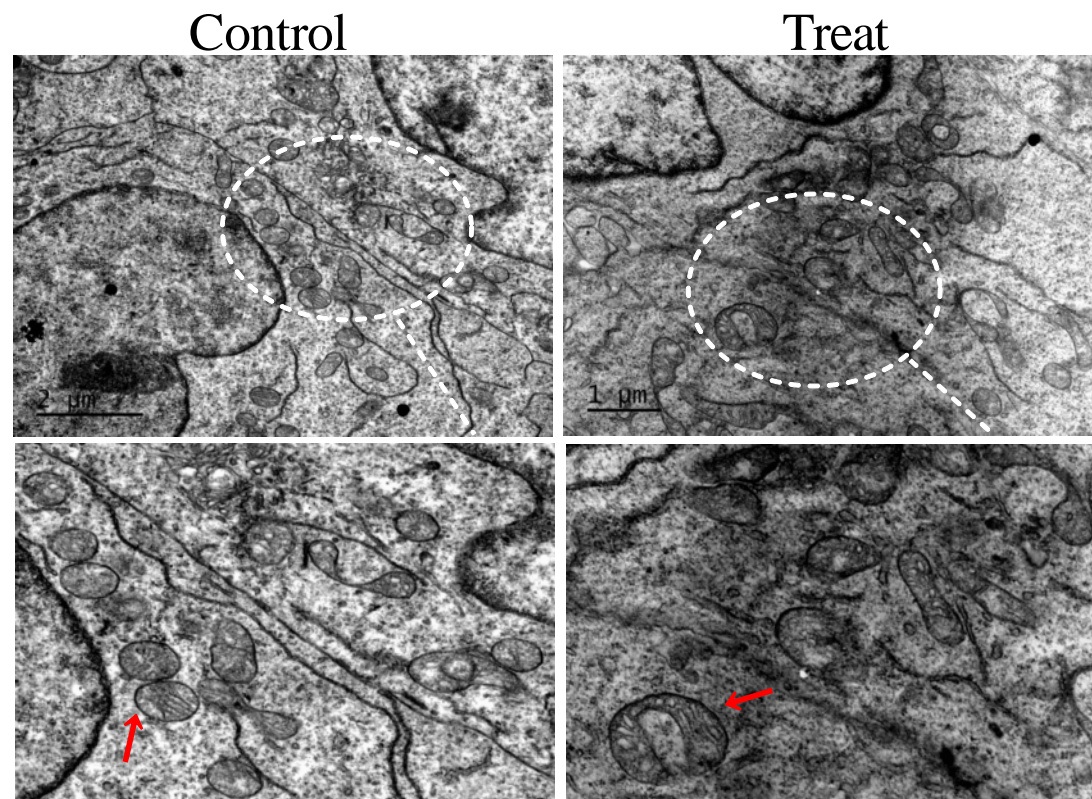

(b)

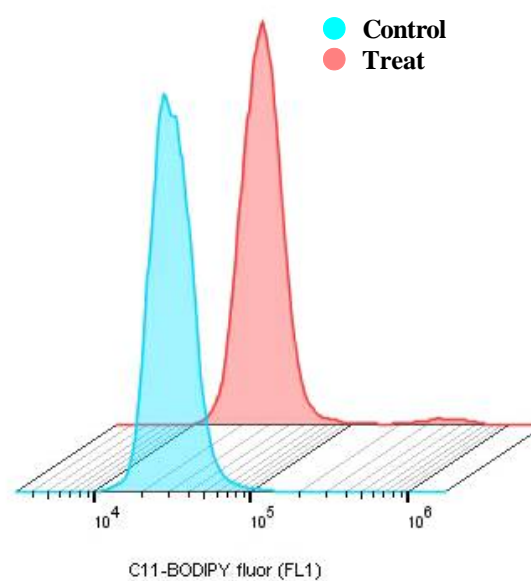

(c)

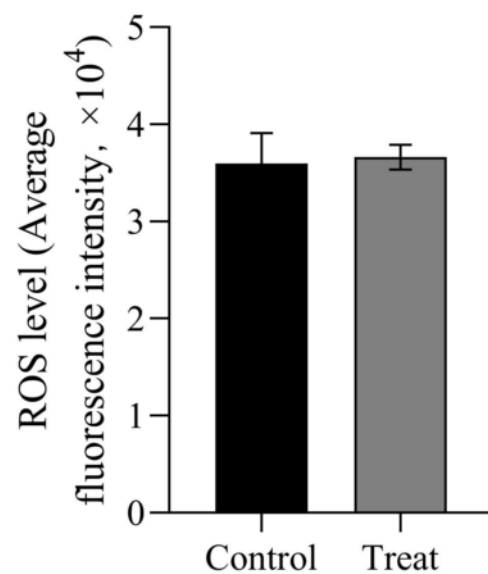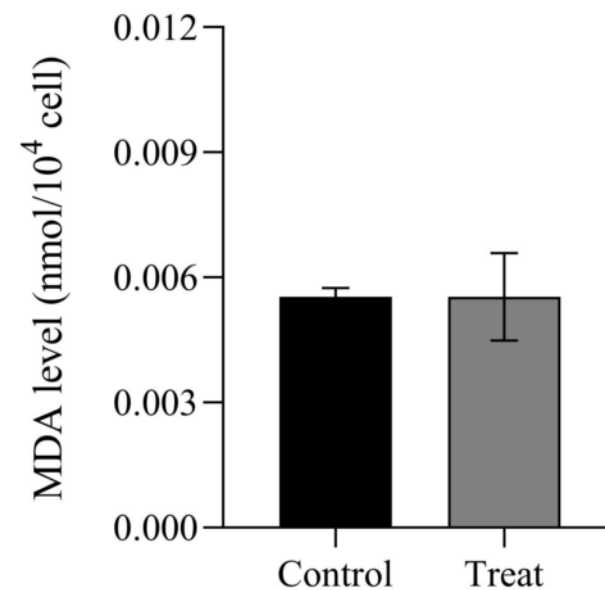

(d)

Supplement: Supplementary Materials — Supplementary Figure 1: cysteine depletion cannot induce ferroptosis in K562 cells. (a) K562 cells were treated with Fer-1 (2 μM), necrostatin-1s (10 μM), Z-VAD-FMK (10 μM), or chloroquine (25 μM) cultured in normal or cysteine depletion condition for 24 h. Cell viability was measured using the MTS kit. (b) TEM observed the cell ultrastructure changes of K562 cells cultured in normal or cysteine depletion condition for 24 h (scale bar = 1 μm). (c) Flow cytometry tested the ROS level of K562 cells cultured in normal or cysteine depletion condition for 24 h. (d) The levels of MDA were tested in K562 cells cultured in normal or cysteine depletion condition for 24 h. [file 7674565.f1.pdf]
